# Concepts and applications of digital twins in healthcare and medicine

**DOI:** 10.1016/j.patter.2024.101028

**Published:** 2024-08-09

**Authors:** Kang Zhang, Hong-Yu Zhou, Daniel T. Baptista-Hon, Yuanxu Gao, Xiaohong Liu, Eric Oermann, Sheng Xu, Shengwei Jin, Jian Zhang, Zhuo Sun, Yun Yin, Ronald M. Razmi, Alexandre Loupy, Stephan Beck, Jia Qu, Joseph Wu, International Consortium of Digital Twins in Medicine

**Affiliations:** 1 National Clinical Eye Research Center, Eye Hospital, Wenzhou Medical University, Wenzhou 325000, China; 2 Institute for Clinical Data Science, Wenzhou Medical University, Wenzhou 325000, China; 3 Institute for AI in Medicine and Faculty of Medicine, Macau University of Science and Technology, Macau 999078, China; 4 Department of Biomedical Informatics, Harvard Medical School, Boston, MA 02138, USA; 5 Department of Big Data and Biomedical AI, College of Future Technology, Peking University, Beijing 100000, China; 6 Cancer Institute, University College London, WC1E 6BT London, UK; 7 NYU Langone Medical Center, New York University, New York, NY 10016, USA; 8 Department of Chemical Engineering and Nanoengineering, University of California San Diego, San Diego, CA 92093, USA; 9 Department of Anesthesia and Critical Care, The Second Affiliated Hospital and Yuying Children’s Hospital, Wenzhou Medical University, Wenzhou 325000, China; 10 Institute for Advanced Study on Eye Health and Diseases, Wenzhou Medical University, Wenzhou 325000, China; 11 Faculty of Business and Health Science Institute, City University of Macau, Macau 999078, China; 12 Zoi Capital, New York, NY 10013, USA; 13 Université Paris Cité, INSERM U970 PARCC, Paris Institute for Transplantation and Organ Regeneration, 75015 Paris, France; 14 Cardiovascular Research Institute, Stanford University, Standford, CA 94305, USA; 15 School of Medicine, University of Dundee, DD1 9SY Dundee, UK

## Abstract

The digital twin (DT) is a concept widely used in industry to create digital replicas of physical objects or systems. The dynamic, bi-directional link between the physical entity and its digital counterpart enables a real-time update of the digital entity. It can predict perturbations related to the physical object’s function. The obvious applications of DTs in healthcare and medicine are extremely attractive prospects that have the potential to revolutionize patient diagnosis and treatment. However, challenges including technical obstacles, biological heterogeneity, and ethical considerations make it difficult to achieve the desired goal. Advances in multi-modal deep learning methods, embodied AI agents, and the metaverse may mitigate some difficulties. Here, we discuss the basic concepts underlying DTs, the requirements for implementing DTs in medicine, and their current and potential healthcare uses. We also provide our perspective on five hallmarks for a healthcare DT system to advance research in this field.

## The bigger picture

Recent advances in technology have facilitated the efficient collection and detailed analysis of human health data. The creation of personalized healthcare digital twins (DTs) permits simulations that predict individual health trajectories and disease progression. Using various health metrics, these DTs enable a quantitative analysis of vital life processes, provide dynamic health guidance, and refine strategies for disease treatment. This innovative approach aims to enhance the mathematical understanding of biological mechanisms, transform existing clinical practices, and truly personalize medical care.

## Teaser

In this paper, the authors explored fundamental concepts of digital twins (DTs) with a focus on their application in medicine. They address challenges in the development of healthcare DTs, including technological barriers, ethical issues, and biological diversity. The review highlights current and prospective applications of DTs in healthcare and suggests five essential hallmarks that should be incorporated into any healthcare DT framework.

## Introduction

**Figure 1:**
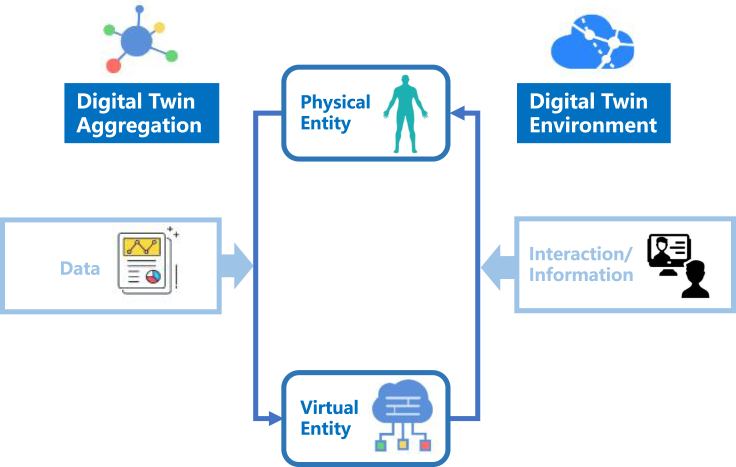
Basics of DTs A virtual entity, a physical entity, and an input data flow for real-time collection and monitoring of the physical entity’s state or physiological functions, along with an output data flow for real-time interaction and communication, such as transmitting diagnosis and treatment solutions.

A digital twin (DT) is a digital and virtual representation of any physical entity. At the core of DTs is a mathematical model that uses data gathered from the physical entity to update the digital counterpart. This iterative approach then allows data to be generated from the digital entity indistinguishable from the physical entity.^1^ DTs are widely used in the engineering and manufacturing industries for monitoring and modeling processes and optimizing efficiency.^2^ Examples include jet engine performance evaluation and the development of smart cities.^3^ In the context of medicine (Figure 1), the physical entity can refer to the patient being studied in their real-world existence, incorporating all molecular, physiological, lifestyle, and environmental information across time.^4^ The virtual entity is, therefore, a digital replica of the patient, or even a virtual space of many digital patients. These digital replicas have characteristics similar to those of the patient, which enable predictions and simulations of the biological processes or disease states using data collected from the patient. The physical and virtual entities communicate via a physical-to-virtual connection to allow continuous update of the parameters that reflect the state of the physical entity.^5^ In essence, a medical DT represents a virtual representation where clinical and medical decisions can be tested before application in the actual patient.^6^ Therefore, this enables the real-time dynamic modeling of biochemical pathways, cells, tissues, diseases, and, ultimately, the entire human body, making personalized medicine a tantalizing reality.

The unique opportunities offered by DTs address the human population as individuals to provide improved and personalized therapies and preventions.^7^^,^^8^ The construction of a DT in medicine will require the integration of diverse information such as clinical data, real-time physiological changes, and the -omics of an individual. For example, DTs may enable the provision of a personalized, on-demand risk profile for chronic diseases, offer lifestyle suggestions to mitigate these risks, deliver warnings about immediate health risks, and provide alerts for pre-emptive diagnostic tests.^9^ Evaluating individual patient responses to a particular drug and forecasting its efficacy and potential adverse effects will also be possible. DTs, therefore, represent a faithful implementation of personalized medicine.^10^^,^^11^^,^^12^^,^^13^^,^^14^^,^^15^^,^^16^

While the application of DTs in healthcare will represent an essential step toward truly personalized medicine, the significant heterogeneity inherent in human populations in genomics, physiology, lifestyles, and environment represents a real and significant hurdle. Significant developments in artificial intelligence (AI), large language models (LLMs), and wearable devices may provide solutions to some of these hurdles. This article provides an overview of the challenges and opportunities offered by these latest developments and their potential integration into a medical DT platform. We then review some examples of DTs already used in healthcare and medicine. Finally, we suggest some critical components for the ideal medical DT.

## Challenges and opportunities to DT implementation in medicine

### Data acquisition

One of the major challenges of a medical DT is the acquisition of sufficient data to make meaningful predictions about the physical entity (the patient). The All of Us research program launched by the U.S. National Institutes of Health in 2018 seeks to gather data from at least 1 million individuals to create one of the largest and most diverse datasets on health and genomics.^17^ Furthermore, next-generation sequencing, high-throughput multi-omics profiling, and mass spectrometry can evaluate the transcriptome, methylome, proteome, histone post-translational modifications, and the microbiome at unprecedented speed and scale.^18^ Nevertheless, the All of Us program focuses on the U.S. population, which may limit any predictive outputs. Therefore, the blueprint of the All of Us program should be established in multiple countries to achieve a truly global and representative cohort.

In addition, to handle large amounts of data, the medical DT should be able to ensure real-time data collection, integration, and interoperability among different platforms and systems. The maintenance of data fidelity is also very important.^19^ For instance, constructing a high-fidelity virtual patient is challenging due to the typically sparse communication rate between the physical and virtual entities compared with mechanistic processes. Therefore, continuous monitoring of static multi-modal health data, including clinical phenotypes and multi-omics such as genomics, metabolism, physiology, and lifestyle parameters, is required. There is also a need for health data standardization to enable data integration and interoperability among different DT providers. Furthermore, advances in biosensor technology have enabled real-time data capture using small, implanted biosensors. Small broadband acoustic and mechanical sensing devices can accurately and continuously measure respiratory airflow, intestinal motility, and other physiological events, such as the cardiac cycle.^20^ Self-sustaining wireless charging using metamaterial surfaces has been explored to enable battery-less pacemakers.^21^ Soft wearable sensors with wireless communication capability will be the next frontier in population health data acquisition. Such wearable digital health technologies are developing rapidly to make previously unavailable data outside of the clinic, such as behavioral and physiological data, available to clinicians so that additional considerations can be considered in clinical decisions and diagnoses.^22^ In addition, facial, fundus, and tongue images can be used to predict underlying pathologies such as cardiovascular disease and diabetes.^23^^,^^24^ These images can be obtained using standard imaging tools. The ultimate application of a medical DT is, therefore, an integration of these multiscale data to observe and predict deviations from the normal state of each individual.^25^ For example, diabetic patients can be provided with customized recommendations on how to improve their health by tracking food consumption, physical activities, and daily life routines. The medical DT platform can also search the virtual world for similar patients to glean peer insights on improving quality of life.

### Building with AI and metaverse

#### Opportunities

To construct a medical DT with high performance in making efficient and inclusive decisions, integrating large-scale AI models into healthcare is necessary. In addition, high-quality datasets are required to train these integrated AI modules.^26^ Data acquired from super cohorts, such as the All of Us program, are ideal for this purpose. Such integrated AI modules can take multi-modal data to make clinical diagnoses,^7^^,^^27^^,^^28^ predict treatment outcomes,^29^^,^^30^ and interpret radiographical images.^31^^,^^32^^,^^33^ DTs created using large multi-modal AI models are more likely to mimic their real-life counterparts. Such approaches have been proposed in oncology,^34^ cardiovascular health,^14^ and neurodegenerative disorders.^15^ Platforms for collecting and providing large amounts of multi-modal data have also been established.^10^^,^^35^

**Figure 2:**
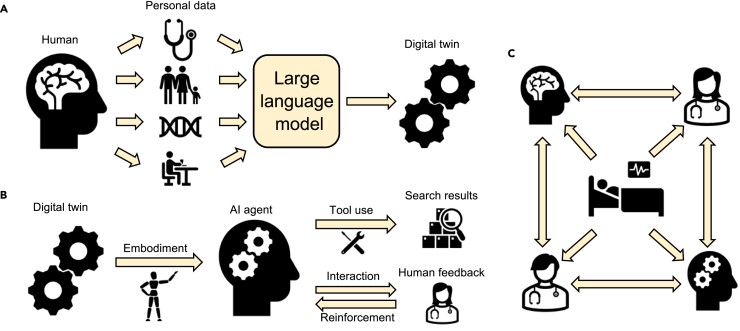
Building with AI and metaverse (A) Building DTs with LLMs. (B) Combining embodied AI with LLM-powered DTs to construct AI agents. (C) Metaverse provides a shared space for physical and virtual entities to communicate regarding patient care.

Recent advances in LLMs, embodied AI, and the metaverse provide exceptional opportunities to make medical DTs a reality.^36^ LLMs refer to deep neural networks trained on vast amounts of text with billions of parameters that can understand and generate human-like text.^37^^,^^38^ Multi-modal LLMs,^28^^,^^39^^,^^40^ which encompass diverse input modes in addition to textual inputs within a unified framework, set the stage for a comprehensive approach to healthcare. Addressing these challenges will require a foundation AI system^41^ that is capable of integration and interpretation of multi-modal data and will output with both holistic and specialist modes. This offers transformative potential for various healthcare scenarios, from answering health questions to clinical diagnostics and mortality prediction.^42^^,^^43^^,^^44^ Leveraging LLMs to improve medical DT models will also lead to better predictions of disease progression and optimized treatment plans (Figure 2A). Through enhanced linguistic competencies, LLMs can convincingly replicate human-like thought patterns and emotional responses.^45^ These models can emulate behaviors, decision-making patterns, and personality traits previously perceived as human by tailoring their interactions based on natural language inputs.^46^ These unique characteristics will allow for customization and personalization,^47^ which align well with the need to construct medical DT models. LLMs can also collaborate with medical DT models to provide dynamic and real-time simulation of physiological processes and patient-specific health conditions. Moreover, the integration of LLMs with DTs can enhance the ability to make informed decisions in scenarios where the underlying mechanisms are not clear.^14^^,^^48^

Embodied AI learns from interactions with environments instead of static datasets^49^ (Figure 2B). In medicine, embodied AI has been playing important roles in mental healthcare^50^ and medical robotics.^51^ One successful story is the development and use of embodied AI robots as DTs of psychotherapists that provide therapy interventions to children with autism spectrum disorders.^52^ The incorporation of LLMs can also seamlessly integrate various models and modalities, as well as orchestrate complex tasks such as planning, scheduling, and collaboration. This will pave the way for the development of versatile, general purpose embodied AI systems.^28^ These LLM-powered embodied AI models are often referred to as AI agents.^53^ Such AI agents can perceive and interpret their surroundings, tackle and resolve problems, and exhibit intelligent behaviors (Figure 2C). This can be achieved autonomously in conjunction with other AI agents or in synergy with human beings. Combining DTs with AI agents may redefine patient care, diagnosis, and treatment.^54^ By leveraging insights gained from human behavior and decision-making processes, AI agents can be used to build virtual patient models, autonomous medical robots, or medical assistants.^12^ For example, a DT of a brain tumor patient undergoing surgery can be built, upon which AI agents can simulate the entire surgical procedure. This enables surgeons to plan the operation by assessing different entry points, angles, and depths, which can decrease the risk of damaging healthy brain tissue. Moreover, the capability of AI agents to orchestrate multiple DT models is important, as complex clinical tasks often require the development of more than one DT.^55^

The metaverse represents a collective virtual shared space created by converging virtually enhanced physical reality, augmented reality, and the internet.^56^ It is a space where digital and physical worlds intersect and open new possibilities in various domains, including medicine.^57^ This raises the possibility that the metaverse could represent a virtual space where healthcare providers and patients can interact with DTs in real-time, allowing for more collaborative and patient-centered care.^58^ For example, doctors could use the metaverse to remotely monitor patients' DTs and adjust their treatment plans based on real-time data. In addition, the metaverse could provide a platform for multidisciplinary teams of healthcare professionals to collaborate and share insights, leading to more informed and effective treatment decisions.^59^ Implementing DTs within a metaverse could also facilitate patient engagement and education, empowering patients to take a more active role in their healthcare.^60^^,^^61^ To build consistent digital models for physical objects in the metaverse,^62^ generative algorithms showed attractive potential. Deep generative models, including the Generative Pre-trained Transformer,^63^ DALL-E (released by OpenAI),^64^ as well as the booming diffusion model,^65^ have been used to create dynamic metaverse environments. Deep generative models have also been extensively used for *de novo* molecular designs, compound optimization and hit identification.^66^^,^^67^^,^^68^

#### Challenges

Although deep learning models have played a key role in solving important problems in computational biology, they are faced with challenges such as interpretability and generalization.^39^ The interpretability of AI is one of the key hurdles to building human trust in a medical DT model, because it requires AI to provide diagnostic or treatment evidence with high transparency and interpretability. Many approaches have been tested in this area. Saliency mapping has been used to demonstrate that networks learn patterns, which agrees with accepted pathological features for Alzheimer’s disease.^69^ The visualization of convoluted neural network ensembles that classify estrogen receptors has also been used to provide interpretability to breast magnetic resonance imaging (MRI) predictions.^70^ Generative discriminative machines^71^ can handle confounding variables to increase confidence in predictions.^71^ Other approaches include interactive learning, causal reasoning, counterfactual reasoning, and mental theory to construct interpretable AI models.^72^^,^^73^^,^^74^^,^^75^

Explainable AI is another challenge. If human intelligence is complemented by AI and sometimes even overruled, we must understand the AI decision-making process. Furthermore, integrating expert knowledge and clinical evidence to guide AI development remains challenging, resulting in some difficulty in revealing the underlying AI explanatory structures. In addition, the AI model in a medical DT platform requires high robustness and generalization when dealing with massive and multisource data. The robustness refers to the tolerance of the model to perturbations in the input data.^76^ Models with poor robustness are easily misled by tiny and simple perturbations in the input.^77^^,^^78^ Many methods for improving model robustness have been applied in the biomedical field, such as an adversarial attack algorithm in gastric cancer subtype analysis models.^79^^,^^80^^,^^81^ Platforms for evaluating the robustness of an AI model have also been proposed.^82^ The degradations in the performance of a model when evaluated on previously unseen data compared with data it has already seen is known as generalization.^83^ Data augmentation using generative adversarial networks can generate a large amount of training data to solve the problems of insufficient data and uneven distribution.^83^^,^^84^ Many generative adversarial networks have been proposed for data augmentation to improve the generalizability of AI models, including CycleGAN,^85^ pix2pix GAN,^86^ and Self-Attention GAN.^87^

The performance of AI systems is known to deteriorate on older tasks during training, which is called catastrophic forgetting. This is a particular issue in implementing medical DTs because continuous learning is necessary. Lifelong learning is a paradigm that allows continuous learning, and to retain prior experience with old tasks while learning new tasks.^88^^,^^89^ Such approaches should be a part of medical DT platform so they can adapt to the real world. Lifelong learning has been explored in the processing and interpretation of medical images,^90^ and elastic weight consolidation has been applied to learning normal brain structure and white matter lesion segmentation.^91^ Reduction of catastrophic forgetting has also been successful in cardiac ultrasound view classification and pneumothorax detection.^92^ It has also been used in dealing with modality and task transitions caused by changes in protocols, parameter settings, or different scanners in a clinical setting.^93^ Research on improving the lifelong learning ability of models will focus on the following aspects: task transfer and adaptation, overcoming catastrophic forgetting, exploiting task similarity, task-agnostic learning, noise tolerance, and resource efficiency and sustainability.^94^

#### Computing power

DT platforms require tremendous computing power.^95^ Quantum computing is well suited to large-scale data processing, information modeling process, and real-world and virtual world communication processes.^96^ In addition, quantum imaging techniques, combined with quantum sensors and quantum dots, are likely to usher in a new era of medical imaging,^97^ and it is expected that quantum MRI machines will produce extremely precise imaging, with the potential to visualize individual molecules.^98^ Combined with AI, quantum computing can also be applied to interpret diagnostic images, identifying anomalies with greater precision than the human eye.^99^ Quantum sensors can also be applied to acquire multi-modal data, particularly in wearable devices, to allow for highly sensitive and accurate monitoring of a physical entity. Quantum dots can be used in conjunction with quantum computing to personalize drug design, potentially enabling tailor-made drugs for each patient, maximizing efficacy and minimizing adverse reactions.^61^ Similar approaches can also be used to develop radiation plans to kill cancer cells without harming healthy cells.^100^ In neurology, quantum computing can simulate complex neural networks, aiding in the understanding and treatment of neurological disorders. This can be applied with DTs of the brain to accurately model the behavior of neurons and synapses, leading to more informed treatment strategies and a better understanding of disorders like Alzheimer’s disease or Parkinson’s disease. Quantum computing and DTs can also be used to optimize hospital operations. Quantum algorithms can analyze patient flow, resource use, and staff scheduling datasets. This enables administrators to optimize the allocation of resources, decrease waiting times and enhance overall operational efficiency in healthcare facilities. Nevertheless, significant developments are needed before quantum methods can be scaled up for these approaches described above. Current challenges include the need for error correction, the stability of qubits, as well as the development of scalable quantum hardware.

#### Accessibility to data

While acquiring data from a large population makes it possible to realize the application of medical DTs, the challenge of data security, privacy, and confidentiality are critical considerations. New rules and regulations prohibit institutions from exchanging medical data without patients’ approval, resulting in the occurrence of data silos. Therefore, creative methods are required to coordinate data retrieval while protecting privacy.^101^ To address these challenges, federated learning (FL) is a promising technology to boost data collaboration across multiple centers rather than sharing raw data. FL sidesteps privacy barriers by allowing clients to update models locally and upload model parameters to the server until the global model gains stable results.^102^ Federated multi-modal learning has been implemented in predicting future oxygen requirements of symptomatic patients with coronavirus disease 2019 (COVID-19).^103^ Cross-silo FL is also an increasingly attractive solution for predicting heart disease hospitalizations through electronic health records,^104^ while cross-device FL has been used to handle continuous health data from wearable devices to deliver personalized health insights.^105^ Swarm learning is another approach that builds a model independently on private data using blockchain technology.^106^^,^^107^ This can track and mediate access to health and genomic records. Additional challenges to accessibility and privacy lie in data heterogeneity, safety, and model communication efficiency. Data heterogeneity can result in client shifts and degrade the convergence of predictive outputs.^108^ In addition, inversion attacks can reconstruct images from model weights or gradient updates with impressive visual details. Poisoning attacks damage the training of global models by deliberately uploading malicious local models, requiring additional privacy-enhancing techniques.^101^ Furthermore, convergence times for FL are limited to communication bandwidths, which affect communication delay times, necessitating the development of communication-efficient FL.^109^

Additionally, the privacy by design approach can enhance data security and privacy at the infrastructure level by implementing robust authentication and access control measures. This includes encrypting data to prevent unauthorized access, using secure protocols like HTTPS or VPNs to protect sensitive data during transmission, anonymizing or pseudonymizing sensitive information, keeping personally identifiable data locally, and establishing a robust system for regular anomaly detection and prevention. Adapting blockchain technology in a medical DT platform can also mitigate the problem of data tampering. The decentralized nature of blockchain technology provides transparency in consent management and allows patients to see who has access and for what purposes.^110^ This will facilitate data audit and allow data changes to be traced. Moreover, self-executing agreements based on predefined rules and conditions called smart contracts can convert physical data governance and regulatory requirements into digital processes. Additionally, tokenization capabilities of blockchain technology can facilitate individual data ownership. In summary, addressing data security, privacy protection, and data ownership is crucial in designing and implementing medical DT technology. This protects sensitive healthcare information, prevents unauthorized access or data manipulation, and fosters trust among stakeholders.^9^^,^^111^

#### Ethics

Several ethical issues related to the extensive collection of sensitive health information arise in any discussions of a medical DT. Therefore, the protection and governance of such collected data are among the top priorities. For example, a determined adversary can hack into a DT repository to potentially harm entire populations. The issue of multiple use of the collected data also needs to be properly addressed and governed, to alleviate the inevitable concern that accumulated sensitive data could be used for purposes other than informing healthcare decisions, such as research, commercialization, or surveillance.

The provision of informed consent will also be critical, particularly with regard to transparency about how the data will be used and who will have access to the data. While the benefits and risks of participating in medical DT projects can be clearly explained to patients in detail, informed consent for collecting individualized information from wearables is more difficult. Furthermore, the extent of multi-modal data involved in the meaningful implementation of medical DTs raises privacy issues and patient confidentiality. One major ethical hurdle is re-identification from anonymized data. This is a particular problem when highly parametrized models such as neural networks are used, as a significant fraction of the training data can be reconstructed from the trained neural network model.^112^ Indeed, a recent systematic review found re-identification rates are high.^113^

Data acquired from medical DT platforms will be used to inform clinical decisions, many of which may be life altering. Where the burden of accountability lies is critically important regarding liability in case of errors or adverse outcomes. In addition, the accumulated data may contain unequal representations of certain demographic groups. This creates a void in the data for machine learning and affects the performance of the medical DT platform with respect to minority groups. The clinical decisions for patients in such groups, or those with a lower socioeconomic status, may therefore contain a certain degree of bias and inequality. Furthermore, it can be envisaged, at least initially, that medical DT technologies will be implemented in settings where the more affluent will benefit. This may exacerbate the inequality gap and bias. The result may be a disproportionate health improvement for high versus low socioeconomic status patients. Unless addressed, these concerns will severely dampen the enthusiasm for the widespread implementation of a medical DT platform.

One additional concern is data ownership. Currently, consent for data provision is received from the data producers, which has led to a digital economy built on centralized data owned by large tech corporations. This system has resulted in a scenario where the data creators (the patients or participants in research) have limited control and oversight of downstream processes. Indeed, tech companies routinely trade and sell personal data for profit. Therefore, in addition to the privacy, ownership, and security issues discussed above, there is also an economic and profit issue that complicates the ethics around medical DT initiatives. Therefore, a fundamentally different approach to a data management philosophy may be needed for medical DT applications. One bold approach may be to empower individuals with full data ownership. This means medical DT applications will provide the data creator the right to keep, sell, donate, or trade their personal data for research or drug discovery. This can be implemented using smart contracts with built-in economic compensation logic. Patients can be compensated when they choose to sell or trade their anonymized data based on their nuanced preference for privacy. Mechanisms to compensate individuals for their health data can also incentivize targeted health data collection for medical research and drug discovery. Such approaches can improve individual digital rights when AI and big data become indispensable components of modern medicine.

## Potential implementations of DTs in medicine

### Health and disease management

#### Individualized homeostasis monitoring

The classification of experimentally or clinically defined normal or healthy states differs slightly in each individual and cannot be extrapolated to large populations.^25^ Currently, treatment personalization relies on low-resolution data and a limited picture of the clinical history of a particular person. For example, there is not yet a clear understanding of a normal blood pressure. The reasons may be due to the relatively sparse blood pressure measurements and the lack of assessment of the impact of physiological and behavioral patterns in any individual.^114^ Without a personalized definition of normal, it is difficult to detect deviations from normal that ultimately constitute the disease state. A medical DT can define normal in each individual through continuous feedback of information between the patient and their DT. Deviations from this normal state define disease, and treatments can be leveraged to predict intervention outcomes.

#### Cancer management

Medical DTs can also realize the promise of precision oncology by integrating individual proteome and clinical data with population data.^115^ Such models continuously learn from new data, as well as individual patient care decisions from physicians, and can be used for real-time adjustment of treatments. This is particularly important in cancer recurrence or drug resistance, and patients may require different surgical, chemotherapy, or radiation regimens depending on the innate resistance of their particular tumor. Medical DT platforms can predict the onset of resistance and offer alternative treatment regimens based on the genome of individual patient tumors. Chemotherapy regimens also can be personalized depending on the patient’s metabolism to mitigate toxic side effects. Such models have shown promise in predicting treatment responses in triple-negative breast cancer.^116^ A medical DT platform can also be used to predict metastatic disease through structured, consecutive radiology reports.^117^^,^^118^

#### Cardiovascular disease

Improved survival and quality of life in cardiovascular disease are achieved by effective acute care and guideline-based risk factor management strategies.^119^ DTs can be created from traditional simulation models and precursor models at different scales to create real-time, cyber-physical systems to provide tailored therapies.^14^^,^^120^ For example, an inverse analytic DT system can detect abdominal aortic aneurysm and its severity scores using neural networks.^121^^,^^122^ The Siemens Digital Heart is used to evaluate the success of cardiac resynchronization therapy by implanting virtual electrodes.

#### Immune responses

A medical DT platform can also play an essential role in autoimmune disorders and infectious diseases. This will require multi-modal, granular, and integrated information at the molecular, cellular, tissue, organ, and body levels. Such platforms can be used to predict the rejection of transplanted organs and the potential responses to immunosuppressive agents. It will also be useful in infectious diseases, particularly during a pandemic, to identify individuals who are susceptible to certain infections or at risk of fatal cytokine storms. It can also be used to predict protective immune responses and immune memory as a result of vaccination.

#### The design, manufacture, and implementation of medical devices

The design of customized medical devices is a great challenge. Creating DTs of different anatomical structures in the body can simplify the design and implementation of customized medical devices. Dassault Systèmes, based in France, developed a model of the structure and function of the human heart using MRI and electrocardiograms. This Living Heart Project has an active collaborative research agreement with the U.S. Food and Drug Administration to evaluate the use of the model in the insertion, placement, and assessment of pacemaker leads, and other cardiac medical devices. Further work from this collaboration will use DT technologies to improve the efficiency of medical devices in clinical trials and leverage simulation data as a source of evidence.

#### Surgery

Surgical interventions offer curative potential for many diseases without effective pharmacological treatment alternatives. However, the surgical procedure and some of the interventions given during the perioperative period may adversely affect patient outcomes. DTs can be very useful in the perioperative period for the planning and simulation of the surgery itself, as well as for predicting surgical outcomes.^123^ A DT platform will also be beneficial for assessing the tolerance of surgery outcomes with respect to small human-induced variations during the surgical procedure, such as during complex heart surgeries like transcatheter aortic valve replacements. Digital orthopedics has generated a DT of the foot and ankle, allowing surgeons to simulate surgery results and optimize surgical planning.

#### Hospital and nursing administration

Outside of the biological and clinical settings, DT technologies can impact the administration of large healthcare institutions. DT platforms created from electronic medical records and live physiological data from wearable devices can be leveraged to provide optimized and personalized medical and nursing services. The Verto Flow platform integrates patient data from various sources using AI algorithms, which healthcare professionals can use to optimize patient care. The ThoughtWire platform can simulate the health status of a patient, alerting doctors when a patient is likely to have a life-threatening complication, and offer suggestions based on predictions to mitigate the risks.^124^ DTs of entire hospital workflows are also being explored by GE and Siemens Healthcare, to optimize surgery schedules and simplify staffing requirements. This can improve overall hospital efficiency and shorten patient waiting times. Project BreathEasy, developed by OnScale, is a DT-inspired lung model to assist clinicians in the prediction of the ventilation requirements in COVID-19 patients. This is particularly important in low-resource regions, where ventilators are in short supply.

#### Synthetic biology circuits

The use of nature in engineering and industry holds immense potential, with synthetic biology emerging as a rapidly growing field with significant economic prospects. Advances in DNA synthesis and sequencing technology have greatly decreased the costs of constructing synthetic DNA genomes. Incorporating advanced microfluidics allows for the creation of cell-free biological components. Indeed, synthetic biology closely aligns with the original concept of DTs. For example, the development of an artificial human heart required substantial DT input.^125^ Incorporating DT technology in synthetic biology could create autonomic biological modules for applications like smart organs, drug production, or renewable energy. These biological modules can communicate with virtual entities on the medical DT platform using synthetic genetic circuits and optogenetic tools. The potential for biological computers suggests a future where control of the virtual entity is integrated within the physical entity. Advances in cellular state estimation and control methods can lead to intelligent designs for biological components, furthering the discovery of new technologies in the control and integration of cellular processes.

## A medical DT: Requirements toward true personalized medicine

**Figure 3:**
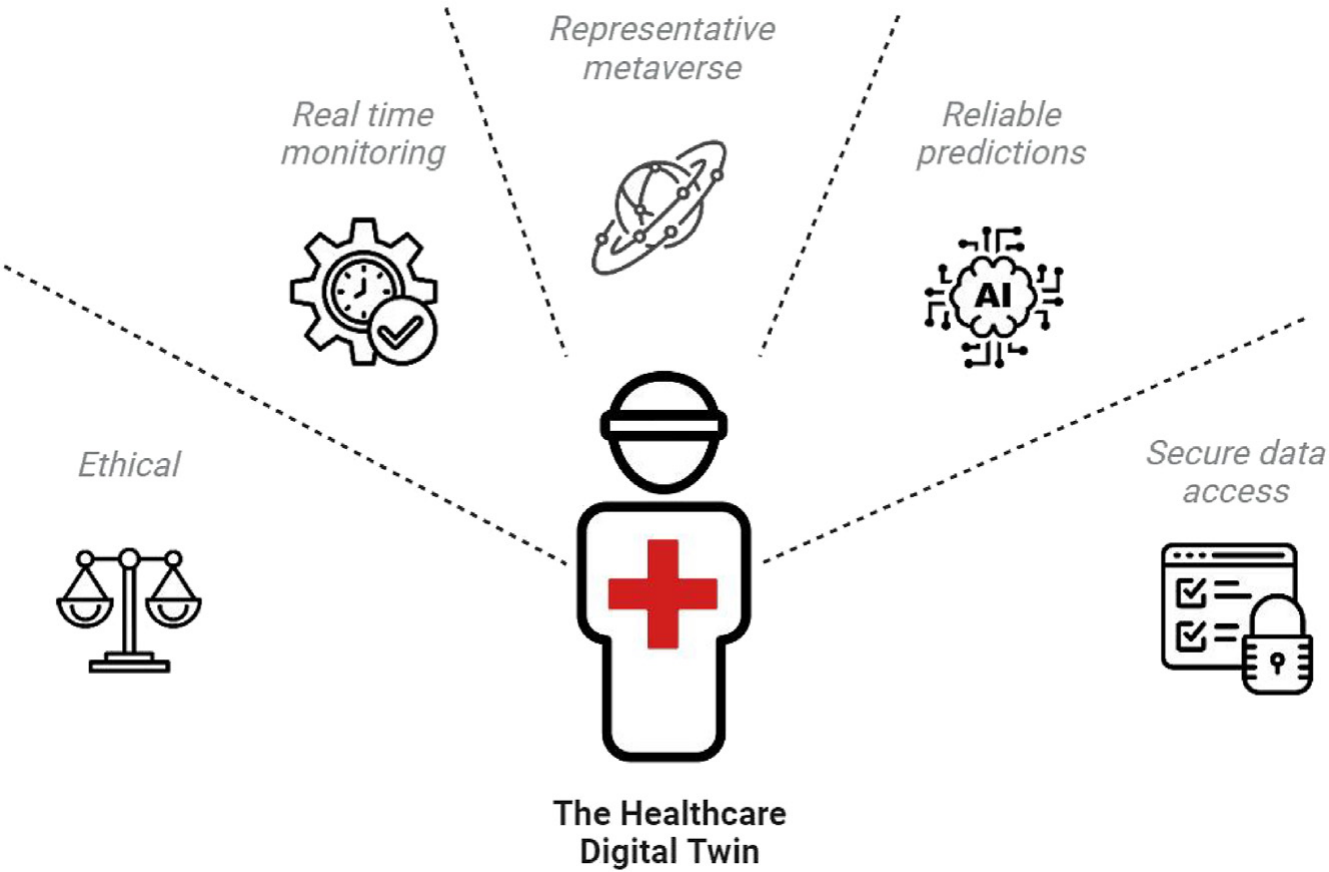
Hallmarks of the DT platform Any healthcare DT should include basic physical-virtual two-way communication, a metaverse of representative data, embodied AI agents based on LLM interfaces, reliable learning and prediction of multi-modal data, real-time patient monitoring, secure data storage, access to patient data, and adherence to ethical standards.

In an era marked by groundbreaking technological advancements, healthcare is on the brink of a revolutionary transformation with the integration of DT technologies. This paradigm shift toward personalized, data-driven healthcare is encapsulated in a medical DT platform characterized by five key hallmarks (Figure 3).

### Hallmarks of a medical DT

#### Representative data repository as a metaverse

**Figure 4:**
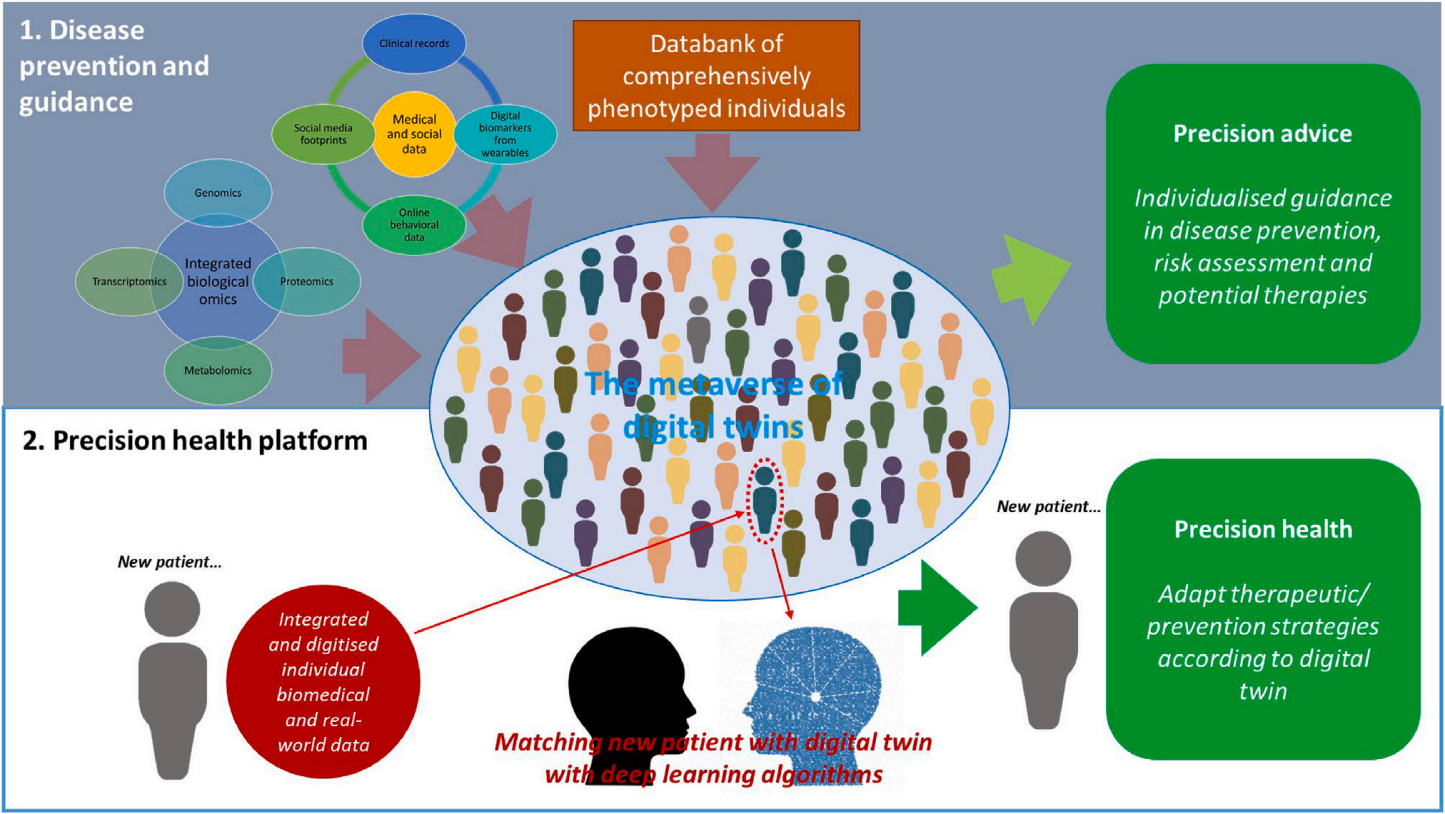
Representative data repository as a metaverse (1) The DT platform integrates extensive multi-modal biological omics and medical data from patients, generating algorithms for individualized guidance in prevention, risk assessment, and therapies. (2) The platform uses comprehensive patient input to match their virtual counterpart in the deeply phenotyped DT database, providing personalized treatment and prevention recommendations.

At the core of a medical DT platform lies the concept of a metaverse, a virtual space of large-scale and high-fidelity digital models and entities that can be used to adjust treatment, monitor response, and track lifestyle modifications (Figure 4). This metaverse should also be where patient-specific DTs and their multi-modal biological omics and medical data coexist, and enables the seamless sharing and interaction of healthcare data. It will also be a dynamic ecosystem that integrates a patient’s comprehensive and multi-modal input to find a matching virtual counterpart and offer individualized treatment and prevention recommendations. The recommendation can be further personalized to the patient based on virtual profile latent-space prototyping if necessary.^126^

#### Real-time monitoring of the physical entity

The second hallmark of a medical DT platform is the real-time monitoring of physical entities. Patient-specific avatars within the metaverse closely mirror patients' real-world health conditions. They can monitor vital signs, physiological parameters, and treatment progress. This real-time monitoring ensures timely interventions and immediate response to anomalies and empowers patients with continuous access to their health data.

#### Reliable predictions by embodied AI agents

Embodied AI agents are the third hallmark of a medical DT platform. They are the digital brains of the platform, constantly analyzing vast amounts of data to offer reliable predictions and insights. These AI agents draw from comprehensive datasets within the metaverse, including historical health data, treatment outcomes, and patient-specific parameters. By simulating different scenarios, they can predict how a patient’s health will evolve, the effectiveness of potential treatments, and the probability of developing specific conditions. This proactive approach transforms healthcare from reactive to predictive, enabling early intervention and optimized treatment strategies.

#### Secure data access

The fourth hallmark of a medical DT platform is a robust emphasis on secure data access, where patient data are protected with the highest security and encryption standards. Patients have control over who can access their data, and healthcare providers are granted secure, role-based access to relevant patient information. This secure data access safeguards patient privacy and complies with stringent data protection regulations.

#### Ethical issues

Ethics is the fifth and perhaps the most vital hallmark of a medical DT platform. It upholds the principle that every healthcare advancement must align with patients' best interests. This platform adheres to the highest ethical data use, research, and care delivery standards. It ensures that patient consent is always obtained and patient rights are respected. The ethical commitment extends to using healthcare data to improve patient care and the broader healthcare community while maintaining transparency and trust.

### Development of a medical DT platform

**Figure 5:**
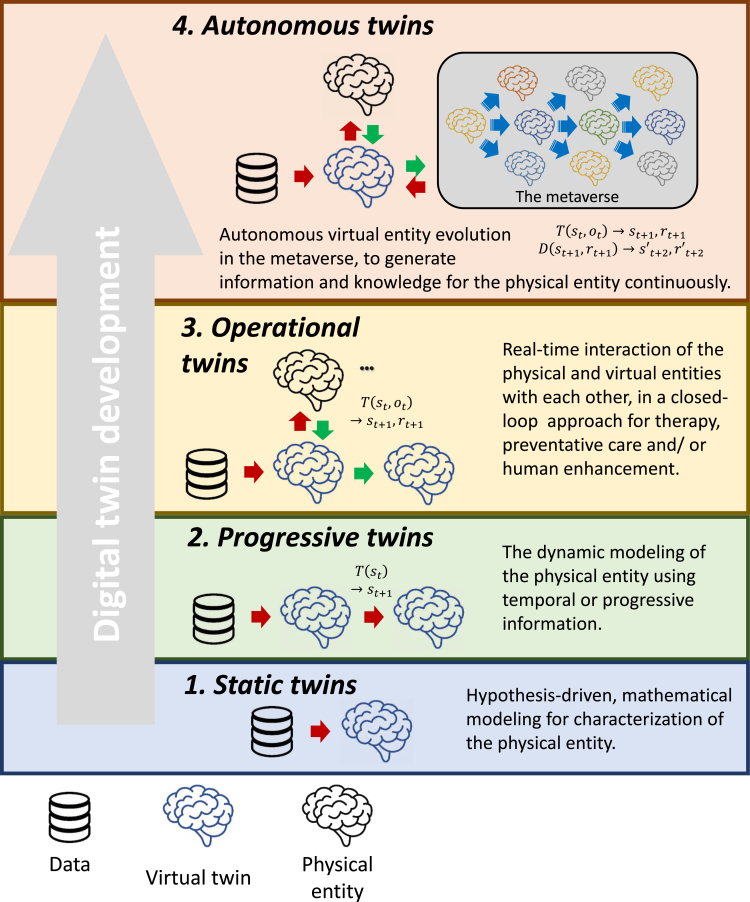
Development of the DT platform Static twins serve as the starting point, where physical entities are digitized, enabling periodic updates to their virtual counterparts. By integrating temporal or progressive information, progressive twins can reflect the evolution of the physical entity and reliably forecast future state transitions. With the development of a closed-loop, iterative improvement framework, operational twins enable real-time interaction between physical and virtual entities. This facilitates both a deeper understanding of biological phenomena and the achievement of specific design objectives in biology and healthcare. In the final stage, the digitized physical and virtual worlds merge, representing the highest level of physical-virtual co-existence, or autonomous twins. Autonomous virtual entities continuously generate information and knowledge for their associated physical entities within the DT platform.

The medical DT platform can be realized using a four-stage development roadmap based on increasing functionality and complexity (Figure 5).

#### Stage 1: Static twins

The simplest DT model starts with a patient model template based on retrospective data and a continuous learning process. The static twin is a traditional simulation and modeling exercise, where analysis is primarily performed offline and characterized by hypothesis-driven mathematical modeling. The static twin is obtained by modeling the state of a physical system through data collected from sensors.^127^ Static twins can, therefore, be considered as data-driven mathematical models of patients. One example of static twins is the HeartNavigator developed by Philips.^128^

#### Stage 2: Progressive twins

The next step in the evolution of the DT platform should incorporate observational data to represent the patient’s current state and reliably forecast future state transitions. It will need existing techniques for simulation, model inference, data assimilation, and high-performance computing to build and test real-time, dynamic models on relatively large scales. Progressive twins integrate temporal or progressive information to construct a dynamic statistical machine learning model, which reflects the evolution of the physical entity and reliably forecasts future state transitions. Progressive twins are, therefore, *in silico* representations that dynamically reflect molecular, physiological, and disease states across time (e.g., aging). An example of progressive twins is the development of three-dimensional brain organoid cell culture models to recapitulate various aspects of human brain physiology *in vitro* and replicate basic disease processes of Alzheimer’s disease, amyotrophic lateral sclerosis, and microcephaly.^129^

#### Stage 3: Operational twins

One of the critical features of the DT concept is the physical-to-virtual connection. Operational twins are real-time, cyber-physical systems that use a continuous connection to monitor state changes in the physical environment. Therefore, operational twins represent a real-time interaction between the physical and virtual entities in a closed loop. For example, an automated insulin injector can be built where changes in the DT of the patient’s blood (data from glucose monitor) can be continuously monitored to determine accurate insulin dose injections throughout the day instead of relying on a fixed injection schedule.

#### Stage 4: Autonomous twins

In the ultimate stage of the DT platform evolution, known as the autonomous twins, the digital and physical worlds are merged, representing the pinnacle of physical-virtual co-existence. The self-sustaining virtual worlds operate independently while interacting seamlessly with the physical world. This integration can create a metaverse populated with countless autonomous, high-resolution virtual entities. For example, an autonomic DT brain could be developed, building on *in silico* representations and evolving autonomously. This can dynamically reflect the biophysical information of an actual brain over time, enabling effective enhancement interventions. Autonomous twins, combined with advanced virtual reality platforms, can also revolutionize surgical practice by providing realistic performance feedback on simulated procedures tailored to each patient.^123^ Autonomous twins can offer valuable insights and guidance for real-world decision-making. The ultimate form of autonomous twins could enable the realization of precision medicine by accelerating the discovery of medical phenomena and disease processes, shortening the timeline for drug discovery, improving surgical outcomes via virtual operations, and simulating disease progression statistics.

### Applications of a medical DT

#### Creation of personalized treatment plans

A medical DT platform can use a cancer patient’s medical history, family history, genetic information, and lifestyle factors (diet, exercise, and exposure to environmental toxins) to create an AI agent. This AI agent, using an LLM, captures the patient’s unique physiological responses and medical conditions,^46^ leveraging external tools for diagnosis and treatment.^130^ It can perform self-diagnosis by accessing the latest medical research, clinical trials, and treatments.^43^ It can consider various treatment options, such as chemotherapy, radiotherapy, immunotherapy, and targeted therapy and assess their potential effectiveness. The AI agent can also analyze the patient’s genetic data to identify mutations or biomarkers that could be indications for or contraindications to specific therapies. The AI agent interacts with the patient and the healthcare provider to address concerns promptly. As new data and feedback are received, the platform uses reinforcement learning to refine its models,^131^ improving the accuracy and effectiveness of personalized treatment plans over time. Once developed, this agent can be easily customized for other patients based on their personal data.

#### Remote patient monitoring

Patients with chronic illnesses, such as hypertension or diabetes, can be equipped with a wearable device that continuously streams their vital signs and health data to their virtual counterparts within the metaverse. The embodied AI agent can analyze the incoming data to detect any irregularities or signs of deterioration. If a critical situation arises, the system can alert the patient and/or healthcare provider. It can even initiate a predefined emergency response. This proactive approach to remote monitoring allows patients to maintain their health from the comfort of their homes or anywhere while receiving immediate interventions when necessary.

#### Virtual clinical trials

Clinical trials are essential for testing new medications and treatments. A medical DT can revolutionize clinical trials by simulating patient responses in the metaverse. Researchers can use the DT platform to represent virtual patients with specific conditions and characteristics. These virtual patients are subjected to various treatment regimens, minimizing the risks and ethical concerns associated with actual patients. The embodied AI agents within the DT platform can analyze the treatment outcomes, providing valuable insights into the potential efficacy and safety of the treatments. This approach expedites the drug development process, reduces costs, and accelerates the availability of new therapies to actual patients.

#### Hospital administration

Integrating DT platforms in hospital administration can substantially improve management of healthcare facilities. These technologies create virtual representations of hospital infrastructure, allowing administrators to monitor and manage operations in real time. For instance, a virtual operating room can provide insights into equipment use, maintenance needs and staff workflows, optimizing space layout and resource use. Clinical workflows and administrative processes can be analyzed and optimized within the virtual environment. Nursing administrators can simulate scenarios to identify bottlenecks and streamline workflows. Virtual entities of nursing staff offer real-time insights into availability, skills, and workload. The DT platform can also simulate patient flows to optimize bed management and predict congestion points. Additionally, the metaverse serves as a training ground for medical staff, allowing them to practice critical care, patient interactions, and new technologies in a risk-free environment.

## Conclusions

The DT concept has proven invaluable in industrial applications, from manufacturing to the safe operation of complex systems. Its potential in developing *in vitro* and *in vivo* research models is also evident in biomedical research. However, its most transformative application lies in clinical medicine, where DT technologies could realize personalized medicine. By combining high-throughput genetic and molecular approaches, single-cell and whole-genome sequencing, big data, cloud-based electronic medical records, and AI, DTs can deliver modern healthcare.

Beyond offering personalized treatment regimens, DT technologies can monitor and predict adverse drug reactions or interactions. Their greatest impact, however, will be in the day-to-day health monitoring of individuals. The ability to precisely predict health perturbations and provide mitigation suggestions will advance the detection and diagnosis of chronic, non-communicable diseases.

Despite the potential, implementation faces obstacles such as privacy issues, data security, and the risk of malicious attacks. Additionally, the accessibility of AI findings and interpretations needs improvement. Nevertheless, the Consortium believes the transformative potential of DTs in healthcare is too significant to be hindered by these challenges. We urge national scientific policymakers and funding bodies to increase resources in this crucial area of research.

## Consortia

Members of the International Consortium of Digital Twins in Medicine include Daniel Baptista-Hon, Stephan Beck, George Church, Wei Gao, Yuanxu Gao, Shengwei Jin, Xiao Liu, Xiaohong Liu, Alexandre Loupy, Eric Oermann, Jia Qu, Pranav Rajpurkar, Zhuo Sun, Joseph Wu, Sheng Xu, Yun Yin, Jian Zhang, Kang Zhang, Hong-Yu Zhou, Marinka Zitnik, Jennifer Zhu Scott, and James Zou.
